# PSMC4 promotes prostate carcinoma progression by regulating the CBX3–EGFR‐PI3K‐AKT‐mTOR pathway

**DOI:** 10.1111/jcmm.17832

**Published:** 2023-07-12

**Authors:** Kaifeng Liu, Shengmin Zhang, Yongzhan Gong, Panyan Zhu, Weigan Shen, Qi Zhang

**Affiliations:** ^1^ Department of Andrology Northern Jiangsu People's Hospital Affiliated to Yangzhou University Yangzhou China; ^2^ Department of Andrology, Northern Jiangsu People's Hospital Affiliated Hospital of Nanjing University Medical School Nanjing China; ^3^ Yangzhou University Medical College Yangzhou China; ^4^ Department of Urology Zhejiang Provincial People's Hospital Hangzhou China

**Keywords:** CBX3, PI3K‐AKT‐mTOR pathway, prostate carcinoma, PSMC4

## Abstract

Proteasome 26S subunit ATPase 4 (PSMC4) could regulate cancer progression. However, the function of PSMC4 in prostate carcinoma (PCa) progression requires further clarification. In the study, PSMC4 and chromobox 3 (CBX3) levels were verified by TCGA data and tissue microarrays. Cell counting kit‐8, cell apoptosis, cell cycle, wound healing, transwell and xenograft tumour model assays were performed to verify biological functions of PSMC4 in PCa. RNA‐seq, PCR, western blotting and co‐IP assays were performed to verify the mechanism of PSMC4. Results showed that PSMC4 level was significantly increased in PCa tissues, and patients with PCa with a high PSMC4 level exhibited shorter overall survival. PSMC4 knockdown markedly inhibited cell proliferation, cell cycle and migration in vitro and in vivo, and significantly promoted cell apoptosis. Then further study revealed that CBX3 was a downstream target of PSMC4. PSMC4 knockdown markedly reduced CBX3 level, and inhibited PI3K‐AKT‐mTOR signalling. CBX3 overexpression markedly promoted epidermal growth factor receptor (EGFR) level. Finally, PSMC4 overexpression showed reverse effect in DU145 cells, and the effects of PSMC4 overexpression on cell proliferation, migration and clonal formation were rescued by the CBX3 knockdown, and regulated EGFR‐PI3K‐AKT‐mTOR signalling. In conclusion, PSMC4 could regulate the PCa progression by mediating the CBX3‐EGFR‐PI3K‐AKT‐mTOR pathway. These findings provided a new target for PCa treatment.

## 1 BACKGROUND

Prostate carcinoma (PCa) is the most common malignancy in men, and is the second leading cause of cancer death in men, and the incidence of PCa increases with age, ethnicity and geographical location.^1^ In addition, lifestyle factors, such as diet and physical activity, may also play a role in the development of PCa. Screening for PCa with prostate‐specific antigen testing is controversial, with some experts recommending routine screening for men over age 50 and others advocating for a more individualized approach based on a patient's risk factors and preferences.^2^ The pathogenesis of PCa has not been identified, which may be related to genetics, environment, sex hormones. Therefore, exploring underlying molecular mechanism and effective therapeutic targets of PCa are warranted.

Proteasome 26S subunit ATPase 4 (PSMC4) is a subunit of the 26S proteasome, which can regulate proteasome assembly,^3^ obesity.^4^, ^5^ The 26S proteasome is composed of two subcomplexes: the 20S core particle (CP) and the 19S regulatory particle (RP).^6^ The 19S RP is directly involved in the recognition and binding of substrate proteins to the proteasome, as well as their unfolding and translocation into the 20S CP.^6^ PSMC4 is a key component of the 19S regulatory particle and acts as an ATPase, providing energy for the unfolding and translocation of proteins into the 20S CP for degradation. PSMC4 is also involved in the regulation of proteasome activity, by controlling the association and dissociation of the 19S RP and 20S CP subcomplexes.^7^ PSMC4 has been shown to be essential for the normal function of the proteasome in a variety of cellular processes, including DNA repair, cell cycle regulation and protein regulation. In addition, PSMC4 can also regulate the progression of multiple types of cancer, including breast cancer,^8^ endometrial cancer,^9^, ^10^ PCa,^11^ oral squamous cell carcinoma.^12^ Studies have showed that high level of PSMC4 was positively correlated with shorter survival in breast cancer.^8^ Moreover, PSMC4 can serve as one of the best reference genes for type 1 endometrial cancer.^9^ But function and mechanism of PSMC4 in PCa remains unclear.

In the present study, we verified the PSMC4 expression in PCa tissues, and clarified the function of PSMC4 in PCa in vivo and vitro. Moreover, we identified chromobox 3 (CBX3) as a downstream target of PSMC4, and regulate PCa progression through epidermal growth factor receptor (EGFR)‐PI3K‐AKT‐mTOR pathway. Therefore, the study elucidated the function of PSMC4, and provided a potential treatment target against PCa.

## 2 MATERIALS AND METHODS

### 2.1 Immunohistochemistry

Tissue microarrays (TMAs) were obtained from Shanghai Outdo Biotech Co., Ltd, and immunohistochemistry (IHC) was performed according a previous study.^13^ In brief, tissue samples were treated, and then incubated with PSMC4 antibody (1:100, ab196589, Abcam), CBX3 antibody (1:100, ab213167, Abcam) and Ki67 antibody (1:100, ab16667, Abcam) overnight at 4°C. Finally, the samples were stained and imaged after secondary antibodies were incubated for 1 h at 37°C. The scores for IHC were performed according to a previous study.^13^ In brief, the score of staining intensity was determined followed 0 to 3+ points. Positive percentage was recorded followed 0 to 4+ points. The scores of IHC were obtained according to the multiplied intensity and positive percentage scores. Low expression indicated a below average score, whereas high expression indicated an above average score.

### 2.2 Cell culture and transfection

Human prostate epithelial cells (RWPE‐1) and PCa cells (DU145, PC‐3 and LNCap; Shanghai Cell Bank) were cultured. In addition, 293T cells were gained from the American Type Culture Collection. PSMC4 short hairpin RNA plasmid (shPSMC4), PSMC4 overexpression plasmid, CBX3 overexpression plasmid, CBX3 short hairpin RNA plasmid (shCBX3) or negative control (Genomeditech) were transfected using FuGene HD transfection reagent (E2311, Promega) per the protocol used in a previous study.^14^

### 2.3 Quantitative real‐time polymerase chain reaction

Total RNA was obtained, and cDNA was synthesized using a commercial kit (Invitrogen). The expression of genes was verified using SYBR‐Green kit (Thermo Fisher Scientific). GAPDH was used to normalize gene expression. The primers were showed in Table S1.

### 2.4 Western blotting

The protein was separated and transferred, and then incubated with 5% milk for 1 h. The protein was incubated at 4°C overnight using antibodies: PSMC4 (ab139184, Abcam), CBX3 (ab213167, Abcam), tropomodulin 3 (TMOD3, ab157215, Abcam), SUZ12 polycomb repressive complex 2 subunit (SUZ12, ab307891, Abcam), LCK proto‐oncogene (LCK, ab227975, Abcam), beta‐transducin repeat containing E3 ubiquitin protein ligase (BTRC, ab71753, Abcam), PI3 kinase p85 (PI3K, 4292S, CST, USA), phospho‐PI3K (BS‐3332R, Bioss, China), Akt (4685S, CST, USA), phospho‐Akt (4060S, CST, USA), mTOR (2983S, CST, USA), phospho‐mTOR (5536S, CST, USA), EGFR (ab52894, Abcam), Bax (ab32503, Abcam), Bcl‐2 (ab182858, Abcam), Caspase 3 (ab32351, Abcam), Cleaved‐Caspase 3 (C‐Caspase, ab32042, Abcam) and GAPDH (1:3000, AP0063, Bioworld). Then secondary antibody was performed and protein was verified using an imaging system (Tanon 5200, China).

### 2.5 Cell counting kit‐8 assay

DU145 cells with PSMC4 knockdown were seeded on 96‐well plates including 2 × 10^3^ cells and cultured for 1, 2, 3, 4 and 5 days. Then, cell counting kit‐8 (CCK8) with 10‐μL was added to each well for 1 h. Finally, the absorbance of 450 nm was detected.

### 2.6 Cell apoptosis assay

The cells were collected and centrifuged. Then cells were washed and 5 μL PE‐PI and FITC‐Annexin V staining was added. The samples were stored in the dark, and centrifuged to remove the supernatant, and cells were resuspended. Finally, the samples were stained with 5 μL PI, and rate of cell apoptosis in DU145 was analysed.

### 2.7 Cell cycle assay

DU145 cells with or without PSMC4 knockdown were harvested and then centrifuged. Then cells were inculated with 70% ethanol at 4°C for 1 h, and the washed and precipitated once with PBS. Then staining was completed, and finally samples were tested.

### 2.8 Wound healing assay

The DU145 cells with or without PSMC4 knockdown were seeded on a six‐well dish with 3 × 10^4^ cells. Then removing culture insert, and adding serum‐free medium. After 24 h, images were analysed using ImageJ software.

### 2.9 Transwell assay

DU145 cells with or without PSMC4 knockdown including 1 × 10^5^ cells were seeded in upper chamber of six‐well plates, and then lower chamber was covered by 600 μL of medium containing 30% FBS for 24 h. Finally, cells were stained for 5 min and taken photos to calculate the rate of migrating cells.

### 2.10 Xenograft tumour model

BALB/c nude mice were injected subcutaneously with 5 × 10^6^ DU145 cells with or without PSMC4 knockdown. Tumour volumes were measured and calculated. Then animals were sacrificed after 30 days. Finally, tissues were stored for study.

### 2.11 Co‐IP assay

The cells were collected and then lysed for 30 min by RIPA Buffer. Then the supernatant was centrifuged at 13400 *g* for 20 min at 4°C. 1 μg of PSMC4 antibody or CBX3 antibody was added to supernatant, and incubated at 4°C overnight. Then 10 μL protein A beads were added. The beads were slowly shaken and incubated for 2–4 h at 4°C to fully associate the antibody with protein A beads. Centrifuge at 845 *g* for 3 min at 4°C and discard the supernatant. Add 15 μL of 2 × SDS loading buffer and boil for 5 min. Binding proteins were determined by western blotting analysis.

### 2.12 Statistical analysis

Statistical analysis was performed using SPSS 24.0. A two‐tailed Student's *t*‐test was used to assess data. Pearson's chi‐square test was used to analyse distribution differences of variables. *p* < 0.05 was used as statistically significant.

## 3 RESULTS

### 3.1 PSMC4 level was markedly increased in PCa

**FIGURE 1:**
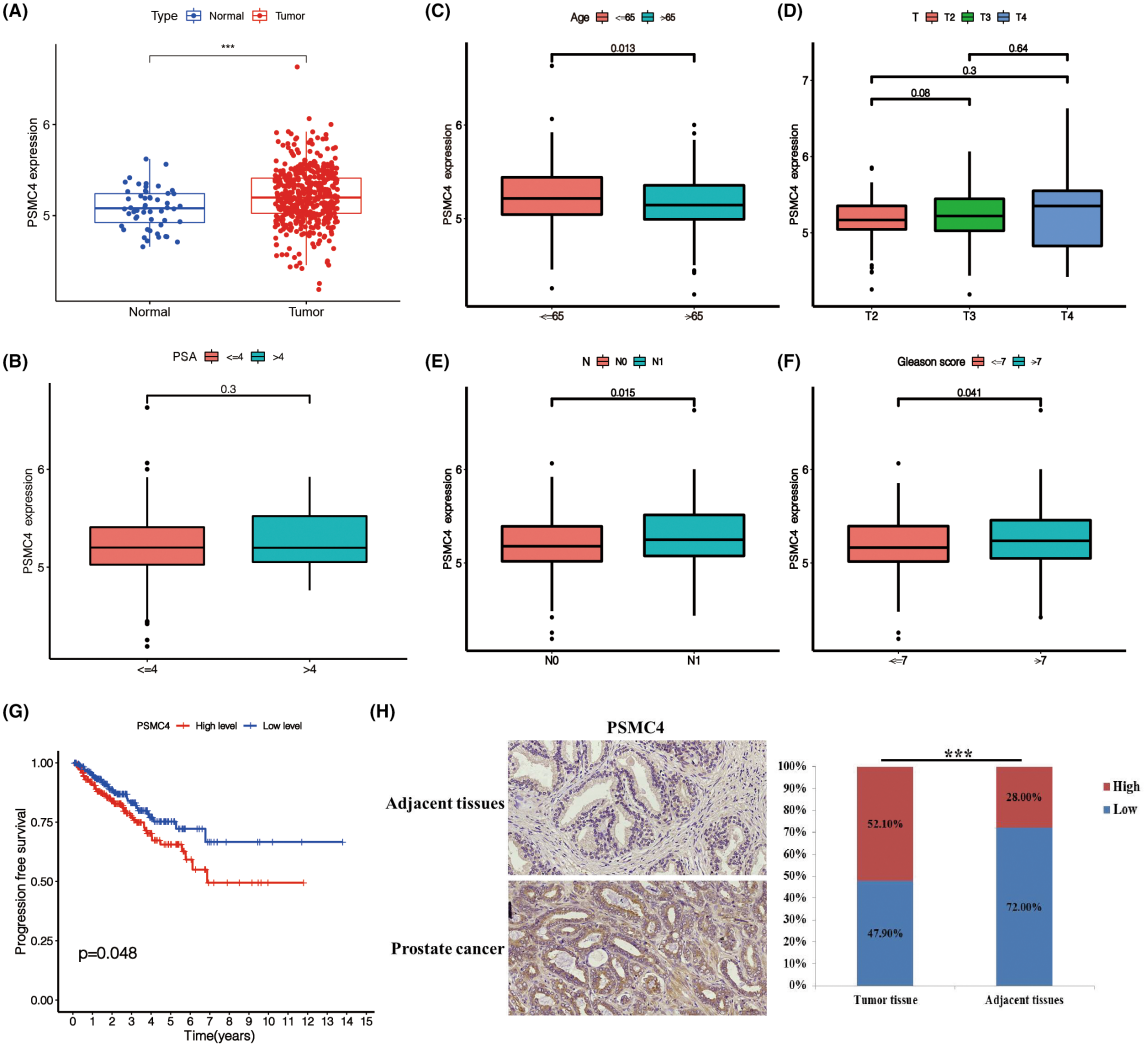
Proteasome 26S subunit ATPase 4 (PSMC4) level was substantially increased in prostate carcinoma (PCa) and associated with poor prognosis. (A) PSMC4 level in PCa were verified through TCGA data; (B) The relationship between PSMC4 level and PSA; (C–F) The relationship between PSMC4 level and Age, Gleason score, TNM stages; (G) Kaplan–Meier overall survival analysis of PSMC4 expression in patients with PCa; (H) Representative images of IHC staining for PSMC4 protein expression, and IHC staining scores of PSMC4. Data are presented as means ± standard deviations. *p < 0.05, **p < 0.01, ***p < 0.001.

To explore the relationship of PSMC4 and PCa, level of PSMC4 was examined. Results showed that PSMC4 level was significantly increased in PCa group compared with the normal group according to TCGA data (Figure 1A). Level of PSMC4 was negatively correlated with age and positively correlated with N stages and Gleason score, but not with T stages and PSA (Figure 1B–F). Moreover, patients with PCa with high PSMC4 level showed shorter overall survival (Figure 1G). Result of TMA showed that the protein level of PSMC4 was significantly higher in the PCa group compared with the normal group (Figure 1H).

### 3.2 PSMC4 knockdown inhibited PCa proliferation and invasion in vitro and in vivo

**FIGURE 2:**
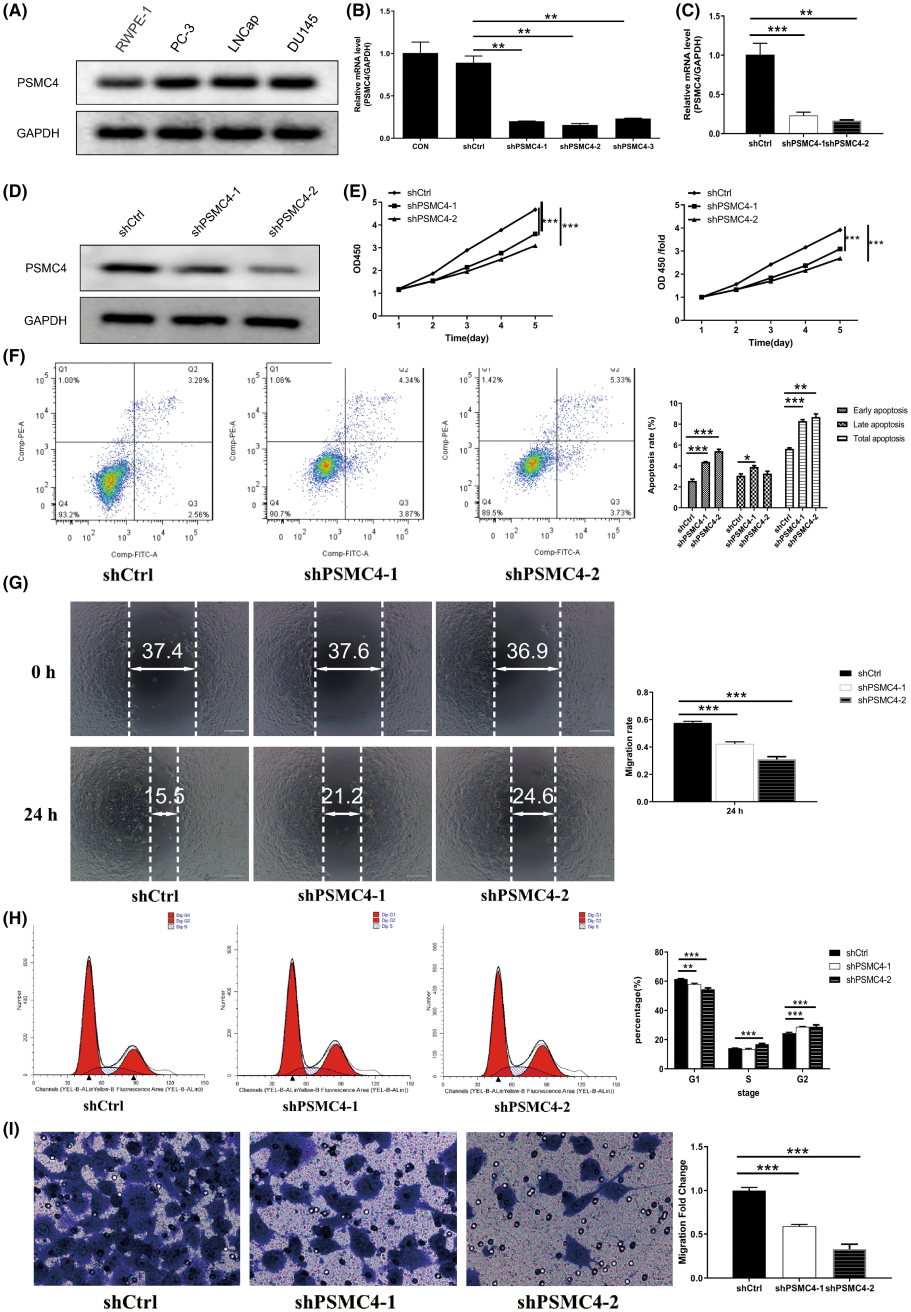
Proteasome 26S subunit ATPase 4 (PSMC4) knockdown inhibited the prostate carcinoma (PCa) progression in vitro. (A) PSMC4 protein level were verified in PCa cells; (B–D) PSMC4 knockdown in DU145 cells were verified; (E) Proliferation of DU145 cells after PSMC4 knockdown; (F) Apoptosis assay, (G) Wound healing assay, (H) Cell cycle assay and (I) Transwell assay were performed after PSMC4 knockdown in DU145 cells. Data are presented as means ± standard deviations. *p < 0.05, **p < 0.01, ***p < 0.001.

To investigate the function of PSMC4 in PCa, we verified PSMC4 expression in RWPE‐1, PC‐3, LNCap and DU145 cells. And result indicated that expression of PSMC4 was significantly elevated in PCa cells (PC‐3, LNCap, DU145), especially in DU145 (Figure 2A). Then PSMC4 knockdown was performed in DU145 cells (Figure 2B–D), and cell proliferation was significantly inhibited after PSMC4 knockdown (Figure 2E). In addition, PSMC4 knockdown significantly induced the apoptosis of DU145 cells (Figure 2F), and markedly inhibited the migration speed of DU145 cells (Figure 2G).

In addition, PSMC4 knockdown markedly reduced the percentage of G1 phase, and increased the percentage of S and G2 phases (Figure 2H), and significantly also inhibited the migration of DU145 cells (Figure 2I).

**FIGURE 3:**
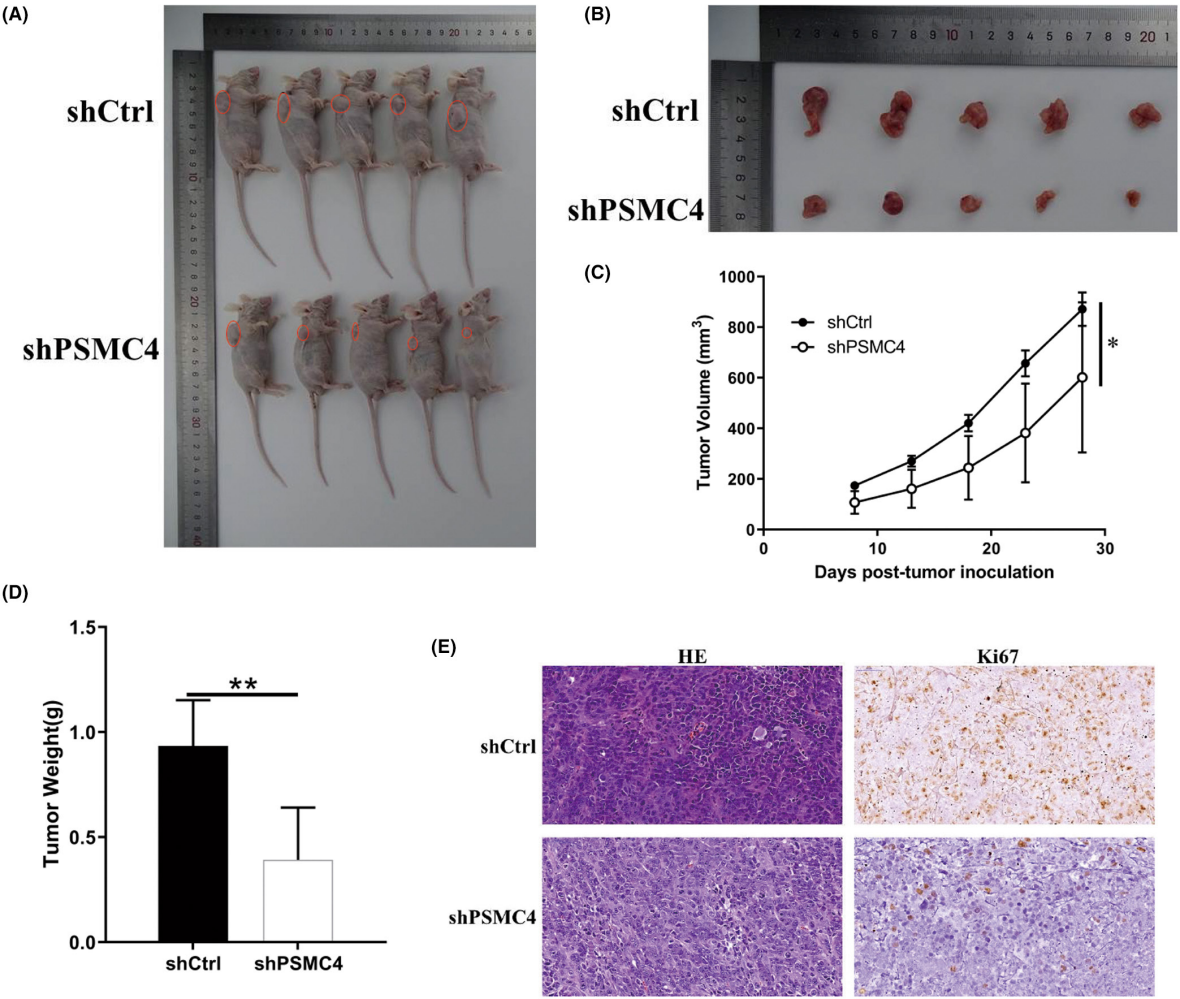
Proteasome 26S subunit ATPase 4 (PSMC4) knockdown inhibited the growth of tumour in vivo. (A, B) Images of tumour were performed; (C, D) Tumour volume and tumour weight were analysed; (E) HE staining and Ki67 expression were showed. Data are presented as means ± standard deviations. *p < 0.05, **p < 0.01.

To further verity the function of PSMC4 in PCa, xenograft tumour model was performed. Results showed that PSMC4 knockdown significantly inhibited growth of tumour and reduced tumour weight (Figure 3A–D). Furthermore, level of Ki67 was significantly reduced after PSMC4 knockdown (Figure 3E). These results indicated that PSMC4 could regulate PCa progression.

### 3.3 CBX3 was a downstream target of PSMC4

**FIGURE 4:**
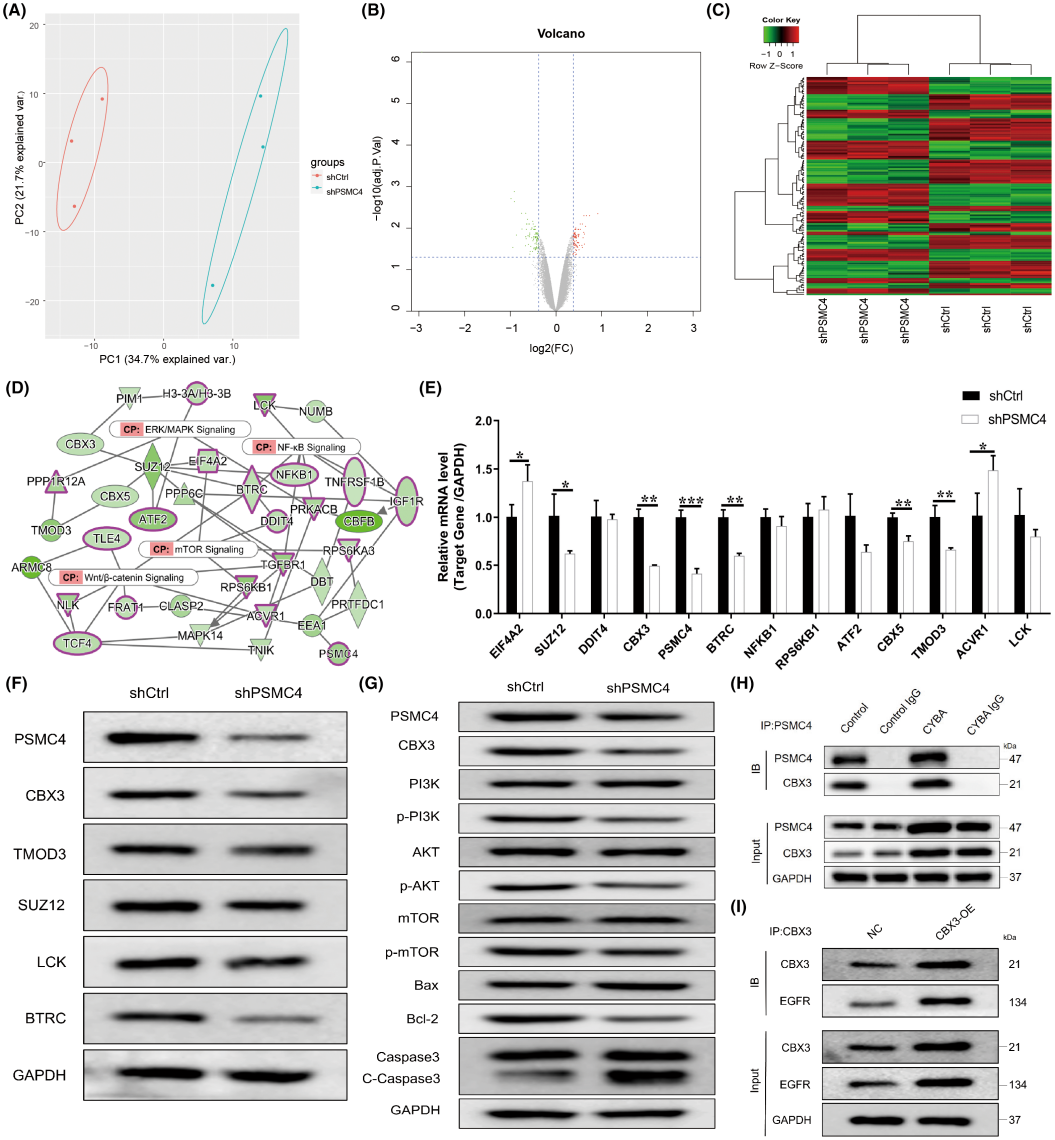
Proteasome 26S subunit ATPase 4 (PSMC4) regulated chromobox 3 (CBX3) expression. (A) Analysis of prostate carcinoma (PCa) was performed; (B, C) Volcano plot and heat map were showed; (D) The interaction network analysis of PSMC4 was performed; (E, F) Levels of DEGs were measured after PSMC4 knockdown; (G) CBX3‐EGFR‐PI3K‐AKT‐mTOR pathway were measured after PSMC4 knockdown; (H) The interaction between PSMC4 and CBX3 was verified by co‐IP assay; (I) The interaction between CBX3 and EGFR was verified by co‐IP assay. Data are presented as means ± standard deviations. *p < 0.05, **p < 0.01, ***p < 0.001.

To explore the potential mechanism of PSMC4 in PCa progression, RNA‐seq was performed. Principal component analysis (PCA) was showed that the trend of separation was obvious between shPSMC4 group and shNC group (Figure 4A). Then 127 differentially expressed genes (DEGs) were found which comprised 61 upregulated DEGs and 66 downregulated DEGs (Figure 4B,C, File S1). Results of interaction network analysis showed that PSMC4 was related to mTOR signalling, NF‐KB signalling and Wnt/β‐catenin signalling (Figure 4D). The levels of DMGs were verified by RT‐PCR, and results revealed that PSMC4 knockdown significantly up‐regulated levels of eukaryotic translation initiation factor 4A2 (EIF4A2) and activin A receptor type 1 (ACVR1), and down‐regulated levels of SUZ12, CBX3, PSMC4, BTRC, CBX5 and TMOD3 (Figure 4E). Moreover, protein level of CBX3 was markedly reduced after PSMC4 knockdown, and there was no difference among other genes (Figure 4F). PSMC4 knockdown also reduced levels of PI3K, AKT and mTOR phosphorylation, and increased levels of Bax and caspase 3 and reduced Bcl‐2 expression (Figure 4G). To further verify the relationship of PSMC4 and CBX3, co‐IP assay was performed, and result showed that PSMC4 directly bond to CBX3, and regulated CBX3 protein expression (Figure 4H). Previous studies showed that CBX3 could regulate EGFR expression, which could promote PI3K/AKT/mTOR pathway.^15^, ^16^, ^17^ To explore how does CBX3 regulate PI3K/AKT/mTOR, co‐IP assay was performed. Result showed that CBX3 overexpression markedly promoted EGFR expression (Figure 4I). These results indicated that PSMC4 may regulate PCa progression by CBX3‐EGFR‐ PI3K‐AKT‐mTOR pathway.

### 3.4 CBX3 level was significantly increased in PCa

**FIGURE 5:**
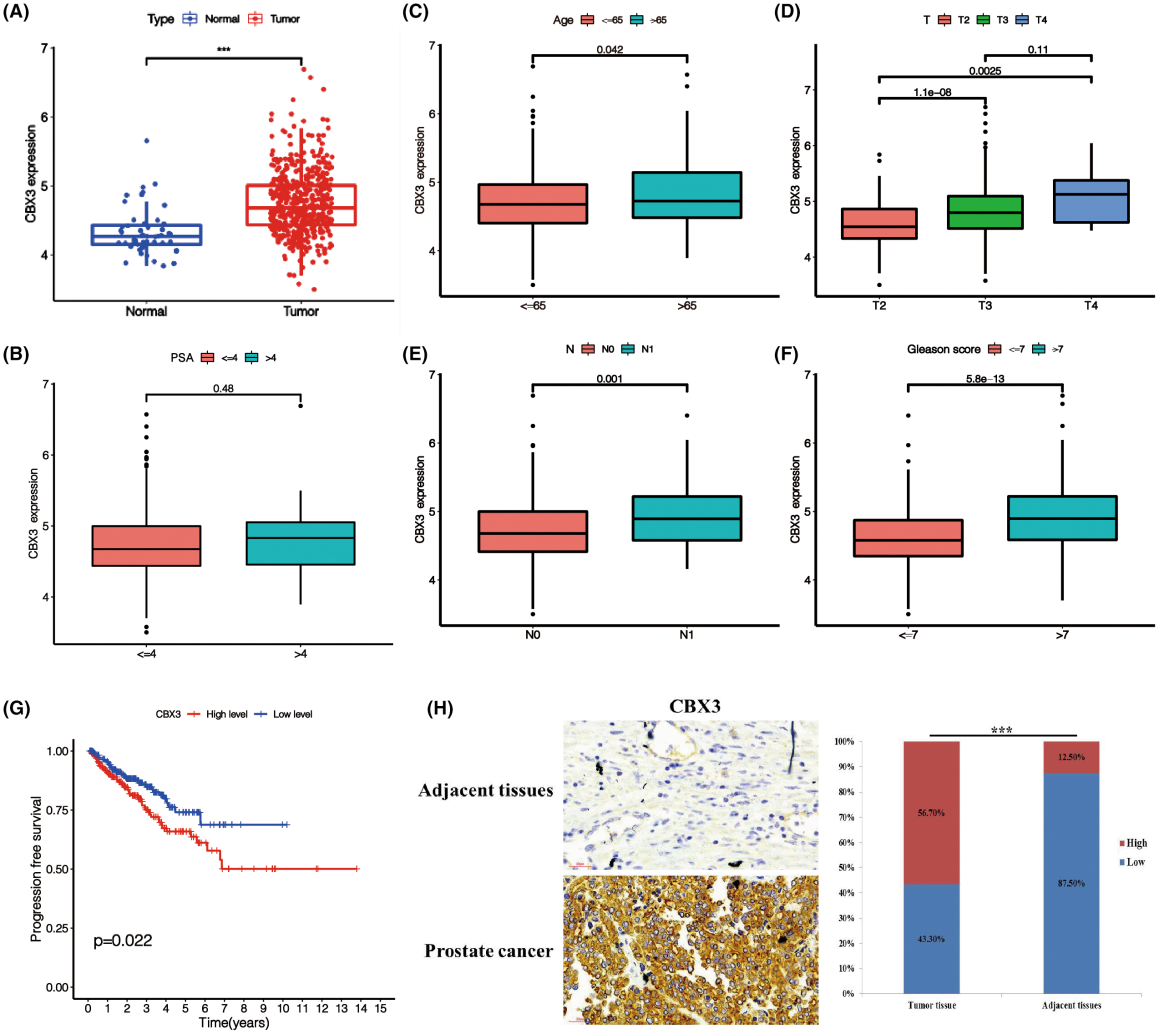
Chromobox 3 (CBX3) level was substantially increased in prostate carcinoma (PCa) and associated with poor prognosis. (A) CBX3 level in PCa was verified through TCGA data; (B) The relationship between CBX3 level and PSA was performed; (C–F) The relationships between CBX3 level and Age, Gleason score, TNM stages were performed; (G) Kaplan–Meier overall survival analysis of CBX3 expression in patients with PCa; (H) Representative images of IHC staining for CBX3 level and IHC staining scores of CBX3. Data are presented as means ± standard deviations. *p < 0.05, **p < 0.01, ***p < 0.001.

CBX3 level was significantly increased in PCa group compared with the normal group according to TCGA data (Figure 5A). And level of CBX3 was positively correlated with age, T stages, N stages and Gleason score, but not with PSA (Figure 5B–F). Moreover, patients with PCa with high CBX3 level showed shorter overall survival (Figure 5G). Result of TMA also showed that the protein level of CBX3 was markedly higher in the PCa group compared with the normal group (Figure 5H).

### 3.5 PSMC4 regulated PCa proliferation and invasion by mediating CBX3

**FIGURE 6:**
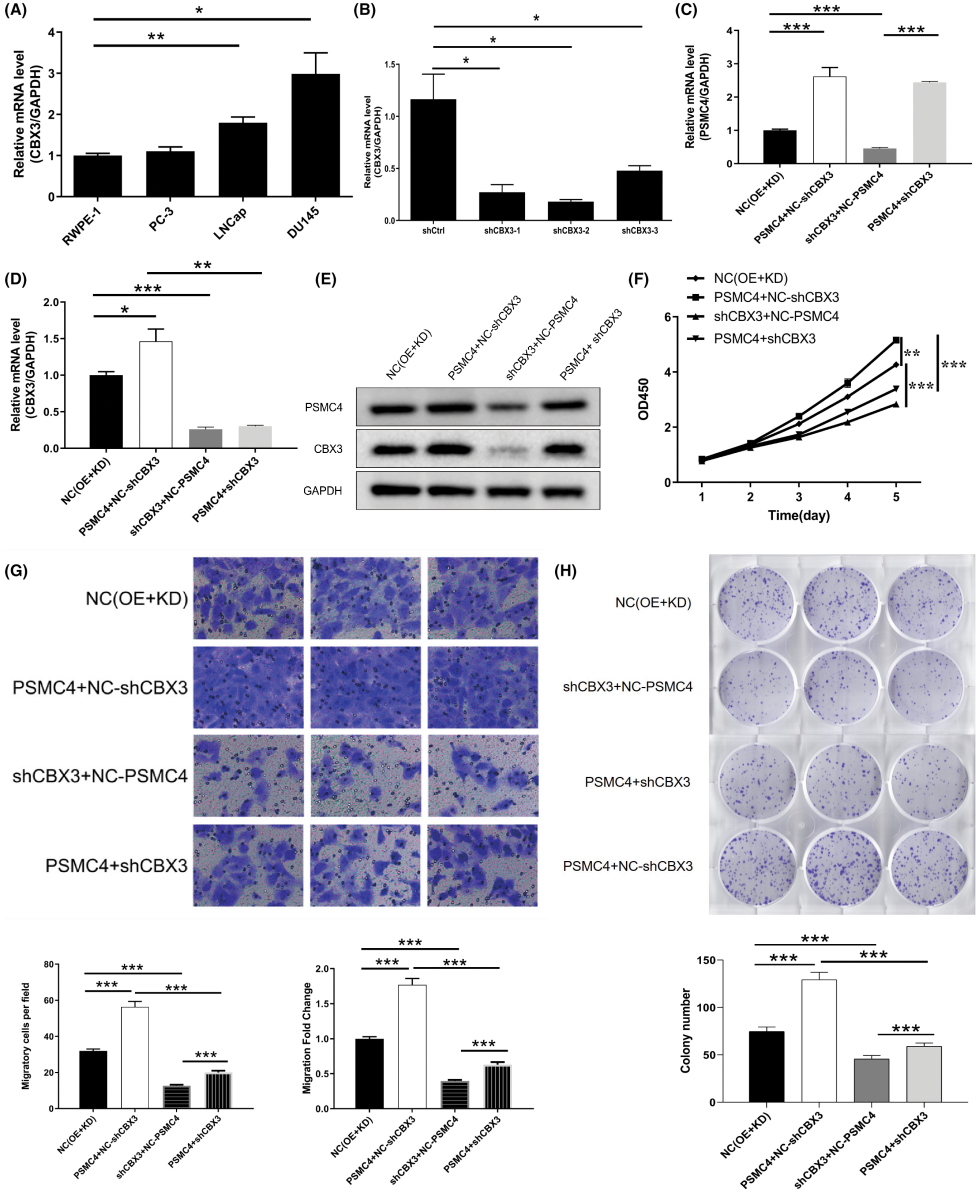
The effect of Proteasome 26S subunit ATPase 4 (PSMC4) on prostate carcinoma (PCa) progression was rescued by chromobox 3 (CBX3). (A) CBX3 mRNA level was verified in PCa cells; (B–E) PSMC4 overexpression and CBX3 knockdown in DU145 cells were verified; The effects of PSMC4 overexpression on (F) cell proliferation, (G) cell migration, (H) clonal formation were rescued after CBX3 knockdown in DU145 cells; (I) The effects of PSMC4 overexpression on EGFR‐PI3K‐AKT‐mTOR pathway were rescued after CBX3 knockdown in DU145 cells. Data are presented as means ± standard deviations. *p < 0.05, **p < 0.01, ***p < 0.001.

CBX3 expression were verified in PCa cells, and result showed that CBX3 level was the highest in DU145 cells (Figure 6A). Then, CBX3 knockdown was verified in DU145 cells (Figure 6B), and DU145 cells with PSMC4 overexpression and CBX3 knockdown were established (Figure 6C–E). PSMC4 overexpression promoted cell proliferation, cell migration and clonal formation in DU145 cells, and the effects of PSMC4 overexpression were rescued by CBX3 knockdown (Figure 6F–H). In addition, PSMC4 overexpression significantly promoted the levels of CBX3, EGFR, Bcl‐2 and PI3K, AKT and mTOR phosphorylation, and inhibited Bax expression. Whereas CBX3 knockdown rescued the effects of PSMC4 overexpression (Figure 6I). These results indicated that PSMC4 regulated PCa proliferation and invasion by mediating CBX3‐EGFR‐PI3K‐AKT‐mTOR pathway.

## 4 DISCUSSION

In the study, we found that PSMC4 level was markedly increased in PCa group compared with the normal group. And level of PSMC4 was negatively correlated with age and positively correlated with N stages and Gleason score. Moreover, patients with PCa with high PSMC4 level showed shorter overall survival. In addition, PSMC4 knockdown markedly induced the cell apoptosis and inhibited cell proliferation, cell cycle and migration in DU145 cells, and PSMC4 knockdown significantly inhibited growth of tumour and reduced tumour weight in vivo. Then PSMC4 overexpression promoted cell proliferation, cell migration and colony numbers in DU145 cells. Mechanistically, PSMC4 knockdown inhibited EGFR‐PI3K‐AKT‐mTOR signalling by reducing CBX3 expression. Finally, the effects of PSMC4 overexpression on cell proliferation, migration and colony numbers were rescued by the CBX3 knockdown.

PCa is the most common malignancy in men, and the incidence of PCa increases with age. Studies have showed that high levels of PSMC4 were positively correlated with shorter survival in breast cancer.^5^ Moreover, PSMC4 can serve as one of the best reference genes for type 1 endometrial cancer.^6^ In the study, we found that level of PSMC4 was increased in PCa, and PSMC4 regulated the progression of PCa in vitro and in vivo, confirming the function of PSMC4 in PCa. Then we further verified that CBX3 was a downstream target of PSMC4, and PSMC4 knockdown reduced CBX3 expression. CBX3 is a component of heterochromatin, and plays important roles in cancers. CBX3 expression was upregulated in gastric cancer, and regulated the malignant phenotype of gastric cancer by mediating chemotherapy and immunotherapy response.^18^, ^19^, ^20^ CBX3 was associated with the poor prognosis and promoted tumorigenesis of osteosarcoma.^21^, ^22^ In addition, CBX3 promoted the progression in hepatocellular carcinoma, and predicted poor survival.^23^, ^24^, ^25^ CBX3 could regulate glycolysis to mediate PCa progression.^26^ In this study, we found that CBX3 was increased in PCa, and patients with PCa with high CBX3 level exhibited shorter overall survival, verifying an important role in PCa. And CBX3 expression was regulated by PSMC4. Previous study showed that CBX3 could regulate EGFR expression, which could promote PI3K/AKT/mTOR pathway.^15^, ^16^, ^17^ In this study, our results indicated that CBX3 promoted EGFR expression, and PSMC4 knockdown inhibited EGFR‐PI3K‐AKT‐mTOR signalling by medicating CBX3, whereas PSMC4 promoted EGFR‐PI3K‐AKT‐mTOR signalling. Studies have verified that PI3K‐AKT‐mTOR signalling could regulate the PCa progression by inducing reprogramming of epithelial to mesenchymal transition, and sunitinib resistance.^27^, ^28^, ^29^, ^30^, ^31^ Even though we demonstrated the function of PSMC4 in PCa, and verified that PSMC4 could regulate CBX3 and PI3K‐AKT‐mTOR signalling, further studies in relationship of CBX3 between EGFR‐PI3K‐AKT‐mTOR signalling are required.

## 5 CONCLUSION

In summary, we demonstrated that levels of PSMC4 and CBX3 were markedly elevated in PCa, and patients with PCa with high PSMC4 or CBX3 level showed shorter overall survival. Then PSMC4 could regulate the PCa progression by mediating the CBX3‐EGFR‐PI3K‐AKT‐mTOR pathway. These findings provided a new target for PCa treament.

## AUTHOR CONTRIBUTIONS

**Kaifeng Liu:** Resources (equal); writing – original draft (equal). **Shengmin Zhang:** Data curation (equal); formal analysis (equal); methodology (equal). **Yongzhan Gong:** Methodology (equal); resources (equal); writing – review and editing (equal). **Panyan Zhu:** Data curation (equal); visualization (equal); writing – review and editing (equal). **Weigan Shen:** Conceptualization (equal); project administration (equal); writing – review and editing (equal). **Qi Zhang:** Funding acquisition (equal); project administration (equal).

## CONFLICT OF INTEREST STATEMENT

The authors declare no competing interests.

## CONSENT FOR PUBLICATION

All authors have agreed to the publication of this manuscript.

## Supporting information


Table S1.

File S1.

## Data Availability

The datasets used and/or analysed during the current study are available from the corresponding author on reasonable request.
