# Supplementary material for: PSMC4 promotes prostate carcinoma progression by regulating the CBX3–EGFR‐PI3K‐AKT‐mTOR pathway
Source: J Cell Mol Med. 2023 Jul 12;27(16):2437–47. doi: 10.1111/jcmm.17832 (PMC10424298; doi:10.1111/jcmm.17832)
Supplement: Supplementary file 1 — Table S1. [file JCMM-27-2437-s001.docx]

**Table S1 The sequence of primers in the study**

| **Primer name** | **Forward primer sequence (5'→3')** | **Reverse primer sequence (5'→3')** |
| --- | --- | --- |
| GAPDH | TGACTTCAACAGCGACACCCA | CACCCTGTTGCTGTAGCCAAA |
| EIF4A2 | TCCTATGTCGCCTTCACT | ACAGACGGGTCATTCCAC |
| SUZ12 | CAAAAGTGCGGCAGAAGAGT | TTATTGGGGTAGGGTAAAGAGC |
| DDIT4 | GACGACGGTTGTGAATGA | TGAGCAAGAAAGTTGGGAT |
| CBX3 | TGGCAGGAACTGGCATTG | GCAGAGGAGTAAGAAGGTGGAA |
| PSMC4 | CGAGCGGCATCTACCTACTGG | TCCTCGCTGGGCTTCTTGC |
| BTRC | CTTTCTGAACCGCTGTCT | TTCTTTGTTGCCTCCTTA |
| NFKB1 | GTCTTAGGGTATAATGGGTC | TGATATGCTATGCTGGTCT |
| RPS6KB1 | AGATGTGCCTGTCCTGTG | ACTGTCTGAGCGGATGAA |
| ATF2 | CTGTCCTCTTAACCCAAAT | ATGTCCTGAATCCTCCAC |
| CBX5 | CTGGACATCCTGGACACCG | GCTTGCCCACCTCGTTGA |
| TMOD3 | TCCGAAGGGAAAGGAATAA | AGCTGCCAGGATGAACTCT |
| ACVR1 | TTCAAATGACCGAAGATG | ACAGGTAGGAGGGATAGG |
| LCK | TTCCCTAGTCGCTTCGTG | TGGTGCCCATAGCCTTCT |
